# A comparative study of clinically used fast Monte Carlo dose engines for proton therapy

**DOI:** 10.1002/acm2.70266

**Published:** 2025-09-23

**Authors:** Sherif M. Gadoue, Narayan Sahoo

**Affiliations:** ^1^ Department of Radiation Physics The University of Texas MD Anderson Cancer Center Houston Texas USA

**Keywords:** CPU, GPU, Monte Carlo, proton therapy

## Abstract

**Background:**

Several fast Monte Carlo (MC) codes have been implemented and used to simulate proton transport and calculate patient doses in proton therapy. The resulting dose is typically compared to full MC codes, rather than other fast MC codes.

**Purpose:**

The primary goal of this study was to compare the gamma pass rates (GPRs) of dose calculation from different fast MC codes to evaluate the accuracy of the computation and modeling among these codes.

**Methods:**

Two GPU codes and one CPU MC code were commissioned to model our clinical proton beamline at University of Texas MD Anderson Cancer Center. The GPU models use single 2D Gaussian models, whereas the CPU model uses a double 2D Gaussian model. For comparative evaluation, 70 cancer patients were randomly selected from our clinical practice, 10 from each of the following treatment sites: head and neck, brain, esophagus, lung, mediastinum, spine, and prostate. The calculated dose was compared with the dose from the verification plan created in the clinical treatment planning system (TPS) using 3D gamma analysis.

**Results:**

The accuracy of dose calculation for all fast MC codes compared very well with the calculation from the TPS for the examined patient plans. GPR for all treatment sites ranged from 96.29% to 99.99%. In general, the double Gaussian model pass rate surpassed the single Gaussian model rate despite a slight accuracy reduction for prostate cases. GPRs for the single Gaussian codes ranged from 96.29% to 99.34%, whereas the double Gaussian model achieved a range of 98.68% to 99.99%.

**Conclusion:**

All commissioned codes we examined demonstrated acceptable 3D GPR across all patients and treatment sites tested. Although the CPU MC code was commissioned using a double 2D Gaussian model, the single 2D Gaussian model used in the GPU codes proved to be sufficiently effective, yielding a high GPR.

## 1 INTRODUCTION

The special characteristics of therapeutic proton beams, such as their finite range and sharp Bragg peak, can result in delivery of a relatively high radiation dose to a tumor while sparing the surrounding healthy tissue.^1^ The dose to organs at risk can be further reduced by lowering range uncertainties, which can be achieved in complex geometries by using Monte Carlo (MC) simulation rather than analytical algorithms.^2^ Several general‐purpose MC codes, such as Geant4,^3^ TOPAS,^4^ MCNP,^5^ FLUKA,^6^ PHITS,^7^ and GATE,^8^ have been developed to provide accurate proton dose calculations through detailed physics modeling. Despite the accuracy of the detailed proton interactions models used in these codes, their prolonged computational times make their use in routine clinical dose computation or calculation checks impractical. Even if the dose computation is distributed among multiple parallel processing units, the associated computational time is still particularly long.^9^

In recent years, researchers have made several attempts to speed up MC dose calculation, while maintaining adequate accuracy levels through simplification and optimization of the algorithmic implementation of radiation simulation. One method used to accelerate dose computation is to employ MC pre‐generated and stored particle trajectories computed for the range of materials and proton energies used in the clinic to calculate the dose by repeating the appropriate tracks in the simulation geometry.^10^ This track‐repeating algorithm is highly amenable to parallelization and can run on central processing unit (CPU)^11^, ^12^, ^13^ or graphics processing unit (GPU)^14^ architectures. Another way to improve dose computation efficiency is to simplify the implementation of physics models used in the simulation and distribute computations among multiple parallel CPUs,^15^ a single GPU, or multiple GPUs.^9^, ^16^, ^17^, ^18^, ^19^, ^20^, ^21^, ^22^

Despite the similarity in the conceptual framework of the general algorithms utilized in the CPU‐ and GPU‐based codes, some differences in the assumptions of particle interactions and implementation aspects exist. For instance, Lee et al.^19^ employed a hash‐table data structure to improve GPU memory efficiency with NVIDIA graphics cards. Also, Maneval et al.^21^ used restricted stopping power formalism, which allows for large steps during the simulation while preserving accuracy. Schiavi et al.^22^ developed a simulation platform that can run on a single CPU, multicore CPUs, one GPU or multiple GPU devices. Furthermore, Tseung et al.^9^ incorporated detailed nuclear modeling of proton‐nucleus interactions in their MC implementation, rather than the simplified Fippel and Soukup^23^ model used in the vast majority of fast MC codes.

In the literature, it is customary to benchmark fast MC implementations against full MC codes such as Geant4, TOPAS, and FLUKA—either in homogeneous/heterogeneous phantoms, or patient CT or both. In general, the dose distribution calculated by fast MC codes agrees well with those calculated by full MC codes.^9^, ^15^, ^18^, ^19^, ^20^, ^21^, ^22^ However, to the best of our knowledge, no studies have compared the results of dose calculation of fast MC codes in relation to each other. This gap is particularly relevant given the current lack of broadly accessible commercial secondary dose calculation solutions tailored for proton therapy, and the resulting urgent need to develop or adopt publicly available alternative tools at the individual clinic level. Therefore, the purpose of this work was to present a comparative study of two GPU MC codes—Monte Carlo code for QUIck proton dose calculation (MOQUI) and Fast paRticle thErapy Dose evaluator (FRED)—and the many‐core Monte Carlo (MCsquare) CPU implementation. For each code, we reported the pass rate for the gamma analysis comparison with both TOPAS and measurements, as well as the results of gamma analysis compared with the treatment planning system (TPS) for 70 cancer patients selected from our clinical practice for routine patient‐specific treatment plan quality assurance.

This paper is organized as follows. An overview of MC algorithms, proton beam model commissioning and dose distribution comparison are presented in Section 2. In Section 3, we report the validation and 3D gamma analysis results for all the examined MC codes. We then highlight the implications of this study and potential future work in Section 4, followed by conclusions and a summary of final remarks in Section 5.

## 2 METHODS

In the following, we give a brief introduction about MC algorithms and methods implemented in MCsquare, MOQUI, FRED and RayStation in Subsections 2.1, 2.2, 2.3, and 2.4, respectively. While in Subsection 2.5, we describe our scanning proton beamline along with its commissioning procedures. Subsection 2.6 outlines the beam modeling validation tests, and Subsection 2.7 presents TPS and fast MC codes benchmarking and experimental validation with measurements. Finally in Subsection 2.8, we give details about the selection process of current clinical patients studied in the current work and the 3D gamma analysis used in the comparison of dose distributions.

### 2.1 MCsquare

The open‐source MC dose engine MCsquare^15^ runs on CPU architectures and computes the energy loss from condensed soft electromagnetic interactions using a tabulated database of stopping powers based on PSTAR,^24^ Geant4, or user‐defined data. Fluctuation of continuous energy loss (i.e., energy straggling) is implemented in MCsquare using a Gaussian distribution with variance described by Bohr^25^ with relativistic correction in thick absorbers. Multiple Coulomb scattering (MCS) is modeled using a Gaussian distribution with variance suggested by Rossi and Greisen^26^ using the random hinge method.^27^, ^28^ In regard to nuclear interactions, proton‐nucleus elastic collisions are simulated using the cross‐section data provided in International Commission on Radiation Units & Measurements (ICRU) Report 63.^29^ Proton‐proton elastic interaction cross‐sections are obtained from the Scattering Analysis Interactive Database (SAID) data analytical fit from 10 to 300 MeV described by Fippel and Soukup.^23^ Like proton‐nucleus elastic collisions, inelastic nuclear interactions are directly sampled from the ICRU Report 63 database. In MCsquare, secondary neutrons are neglected owing to their nonsignificant contribution to the local dose deposition, prompt gammas are produced but not transported, kinetic energy transferred to heavier recoil nuclei is locally deposited, and other charged particles—such as secondary protons, alpha particles, and deuterons, are explicitly simulated.^15^

### 2.2 MOQUI

MOQUI^19^ runs on GPU architecture exclusively on one GPU device. The cross‐section data used in the code are based on Geant4 v10.6.p03. For low‐energy protons (i.e., < 2 MeV), ionization cross‐section data are taken from G4BraggModel, whereas G4BetheBlochModel is used for high‐energy protons having energies > 2 MeV. Energy straggling is sampled from a Gaussian perturbation with variance calculated using Bohr formula.^25^ For each step, the angular deflection caused by MCS is calculated by randomly sampling a Gaussian distribution with zero mean and a variance given by the formula proposed by Rossi and Greisen.^26^ Furthermore, for elastic (proton‐proton and proton‐oxygen) and inelastic (proton‐oxygen) interactions, cross‐section data are obtained from the Geant4 QGSP_BERT_HP physics list. The energy of electrons produced during the simulation is locally deposited, whereas neutrons and photons are neglected. Secondary protons are explicitly simulated in a similar fashion to primary protons. In addition, a hash table is adopted for the scoring system in MOQUI to improve memory utilization efficiency.^19^

### 2.3 FRED

The MC code FRED can run on a multicore CPU architectures, as well as single or multiple GPU cards.^22^ The code uses tabulated stopping power data from PSTAR.^24^ For a series of initial proton energies and step lengths, these tabulated data are interpolated and integrated before running the simulation and stored in a lookup table. The energy straggling probability function is described according to the methodology described by Seltzer and Berger;^30^ for thin absorbers, the energy fluctuation can be sampled from the model developed by Landau^31^ and Vavilov,^32^ whereas a Gaussian distribution is used in the thick absorber regime. MCS is implemented using different approaches; the user can choose from single Gaussian models, such as Highland's formula^33^ or Rossi and Greisen^26^ with Fippel and Soukup^23^ correction or double Gaussian approximation with Rutherford correction. Inelastic interaction cross‐sections are taken from ICRU Report 63,^29^ and the model of nuclear elastic scattering is adopted from Fippel and Soukup^23^ for both proton‐nucleus and proton‐proton interactions. Regarding secondary particles, only the production of secondary protons and deuterons is taken into account; all other interaction products are not considered, and their energy is deposited locally owing to their limited range.^22^ Table 1 presents a comparison of the physics models and algorithmic implementations of MCsquare, MOQUI, and FRED.

**TABLE 1:** A Summary of the key features of the physics models and implementation details of MCsquare, MOQUI and FRED.

Aspect	MCsquare	MOQUI	FRED
Architecture	CPU	GPU (single device)	All (Multicore CPU, single or multiple GPUs)
Stopping power	PSTAR or Geant4	Geant4	PSTAR
Energy straggling	Gaussian	Gaussian	Gaussian, Vavilov for thin absorbers
Multiple coulomb scattering	Single Gaussian with Rossi & Greisen variance	Single Gaussian with Rossi & Greisen variance	Multiple options: Single Gaussian with Highland variance, Single Gaussian with Rossi & Greisen variance, Double Gaussian with Rutherford correction
Elastic proton‐proton	Fippel and Soukup	Geant4	Fippel and Soukup
Elastic proton‐nucleus	ICRU 63	Geant4	Fippel and Soukup
Inelastic proton‐nucleus	ICRU 63	Geant4	ICRU 63
Secondary particles	Neutrons are ignored, photons are produced but not transported, secondary protons, deuterons and alphas are included in the simulation, energy of electrons and recoil nuclei are locally deposited	Neutrons and photons are ignored, secondary protons only are included in the simulation, energy of electrons and recoil nuclei are locally deposited	Neutrons and photons are ignored, secondary protons and deuterons are included in the simulation, energy of electrons and recoil nuclei are locally deposited

### 2.4 RayStation

The GPU MC algorithm in RayStation version 12A (RaySearch Laboratories AB, Stockholm, Sweden) can run on multiple GPU devices.^16^ It uses stopping power data calculated using the Bethe‐Bloch formula,^34^ without the shell and density correction terms. The statistical fluctuation of the energy loss is determined by Bohr^25^ approximation. Using the random hinge approach, MCS is modeled according to Goudsmit and Saunderson formalism.^35^, ^36^ Cross‐section data for inelastic proton‐nucleus interactions are obtained from ICRU Report 63,^29^ whereas the cross‐section data of elastic proton‐proton and proton‐nucleus interactions are derived from the parametrization described by Fippel and Soukup.^23^ Photons, electrons, neutrons, and elements heavier than alpha particles produced during the simulation are not transported, and their corresponding energy is discarded or deposited locally. Other secondary particles, such as protons, alpha particles, and deuterons, are transported and explicitly simulated in a manner similar to those for primary protons.^16^

### 2.5 Machine properties and beam commissioning

The University of Texas MD Anderson Cancer Center Proton Therapy Center synchrotron‐2 (PTC2) is a PROBEAT‐FR system (Hitachi High‐Tech Corporation, Tokyo, Japan) that consists of four dosimetrically matched treatment rooms (T1‐T4). To widen the sharp Bragg peaks, especially for low energies, a range modulation device (i.e., ridge filter) is used in our beamline. The scanning beam magnet location is at 135.0 and 192.5 cm from the isocenter for the x and y magnets, respectively. The dose rate is constant at 480 monitor units/min with 68 nominal proton energies in use. PTC2 has two range shifters, both having a water equivalent thickness of about 4 cm. The regular range shifter can extend to 21.0–39.5 cm with respect to the room isocenter and has a 30 cm × 40 cm inner lateral dimension. On the other hand, the extended range shifter, which has a 13 cm × 17 cm inner dimension, can extend an additional 13.5 cm over the end of the snout to reduce the air gap.

To characterize PTC2 clinical pencil beam model parameters, the procedure described by Gajewski et al.^17^ was followed. For each nominal energy in the beam model parameter library, four parameters must be defined: spot size evolution, beam energy, energy spread, and absolute dose calibration. Regarding spot size evolution, measurements of PTC2 in‐air spot profiles have been performed at five different positions, that is, at room isocenter, and at ±10 and ±20 cm from the isocenter. For MCsquare, a double 2D Gaussian fit was performed to calculate the spot size at the source plane. For MOQUI, a single 1D Gaussian fit was done instead, as it implements only a single Gaussian model. For FRED, the evolution of the spot size can be described using two models: the emittance model (referred to as FEM below) and the simplified sigma squared model (referred to as FSS below). The parameters required for the FSS model are obtained by fitting the squares of the measurement points to a parabolic function, whereas the Twiss parameters for FEM can be obtained using the fit results of the FSS model.

Regarding the energy parameters of the beam model, the iterative procedure starts with specifying initial values of proton beam energy for each nominal energy and a user‐defined number of steps around the initial value. For each step in the range, a value of energy is determined (depending on the initial energy, and number of steps) and used in the simulation in a water phantom to obtain an integral dose distribution (IDD) that is fitted to obtain the proton range. The difference between the simulated range and the corresponding measured value obtained from IDD measurements is calculated. The differences and the determined values of energies are then fitted to a parabolic function to determine the energy value corresponding to a zero value for the difference. A similar procedure is carried out for the energy spread but using the Bragg peak width instead of range, and a final simulation with an increased number of primary particles is run using the optimized energy and energy spread parameters to verify its validity.

Finally, the absolute dose scaling factor is obtained by simulating a monoenergetic 10 cm × 10 cm field, 2.5–mm spot spacing, 0.1 monitor units per spot, and a fixed monitor unit scaling factor in water. For each MC code (MCsquare, MOQUI, FEM, and FSS), the dose at an energy‐specific depth in water is derived from the simulation and compared with measurement values for each energy. The new MU scaling factor is obtained from this ratio. Commissioning of all MC codes follows the same methodology using the same measurements acquired during the facility's initial commissioning process.

### 2.6 Beam parameters validation

The commissioned fast MC codes beam models were then validated by comparing the simulation results of the MC codes with the TPS and measurements performed during PTC2 commissioning for three different monoenergetic beams. 79.2, 160.3, and 216.1 MeV were selected to represent low, medium, and high proton energy ranges. IDD and absolute dose simulations were performed in a water phantom whose surface is located at isocenter, while lateral pencil beam profiles were obtained in air at isocenter to mimic our commissioning measurements.

### 2.7 Patient validation and experimental comparison

For patient validation, seven treatment sites were chosen: head and neck, brain, esophagus, lung, mediastinum, spine, and prostate. A representative case for each site was selected to run full‐scale MC simulation in patient CT using DICOM RT‐Ion interface^37^ integrated in TOPAS MC code and commissioned for our proton beamline. We used the stoichiometric tissue conversion methodology^38^ to obtain the CT calibration required for MC simulations. The simulated dose was compared to the dose calculation of the TPS and each MC code to validate the beam model in heterogeneous media. To experimentally validate MC calculations, the results from the TPS and fast MC simulations were compared with 2D MatriXX measurements (IBA Dosimetry, Germany) for the composite of treatment fields at isocenter.

### 2.8 Patient selection and plan comparison

In this study, 70 cancer patients treated at PTC2 scanning proton beamline were selected for comparative evaluation. Ten patients per treatment site were randomly selected for the study; of the cases with prostate cancer, five had prostate‐only plans, and five had lymph node involvement. For all 70 patients, our routine clinical patient‐specific treatment plan quality assurance workflow was followed. Using a cubic water phantom, a verification plan was created for the clinically approved plan in TPS, and the RT dose DICOM file of the verification plan dose calculated using RayStation MC dose engine along with the RT plan DICOM files were exported. Using Python scripts, an input file for each MC code in this study was generated using the RT plan DICOM file to run the simulation in water. After completing the simulation, the calculated dose for each commissioned MC code was compared with the dose calculated using the RayStation MC dose engine. Instead of performing 2D gamma analysis on individual planes, 3D gamma analysis was used to evaluate the whole dose distribution for each patient. Our criteria for gamma analysis were a 3–mm distance‐to‐agreement, a 3% dose difference, and a dose cutoff of 10%. Additional gamma analysis results using 2–mm distance to agreement and 2% dose difference are provided in the supplementary material.

## 3 RESULTS

**FIGURE 1:**
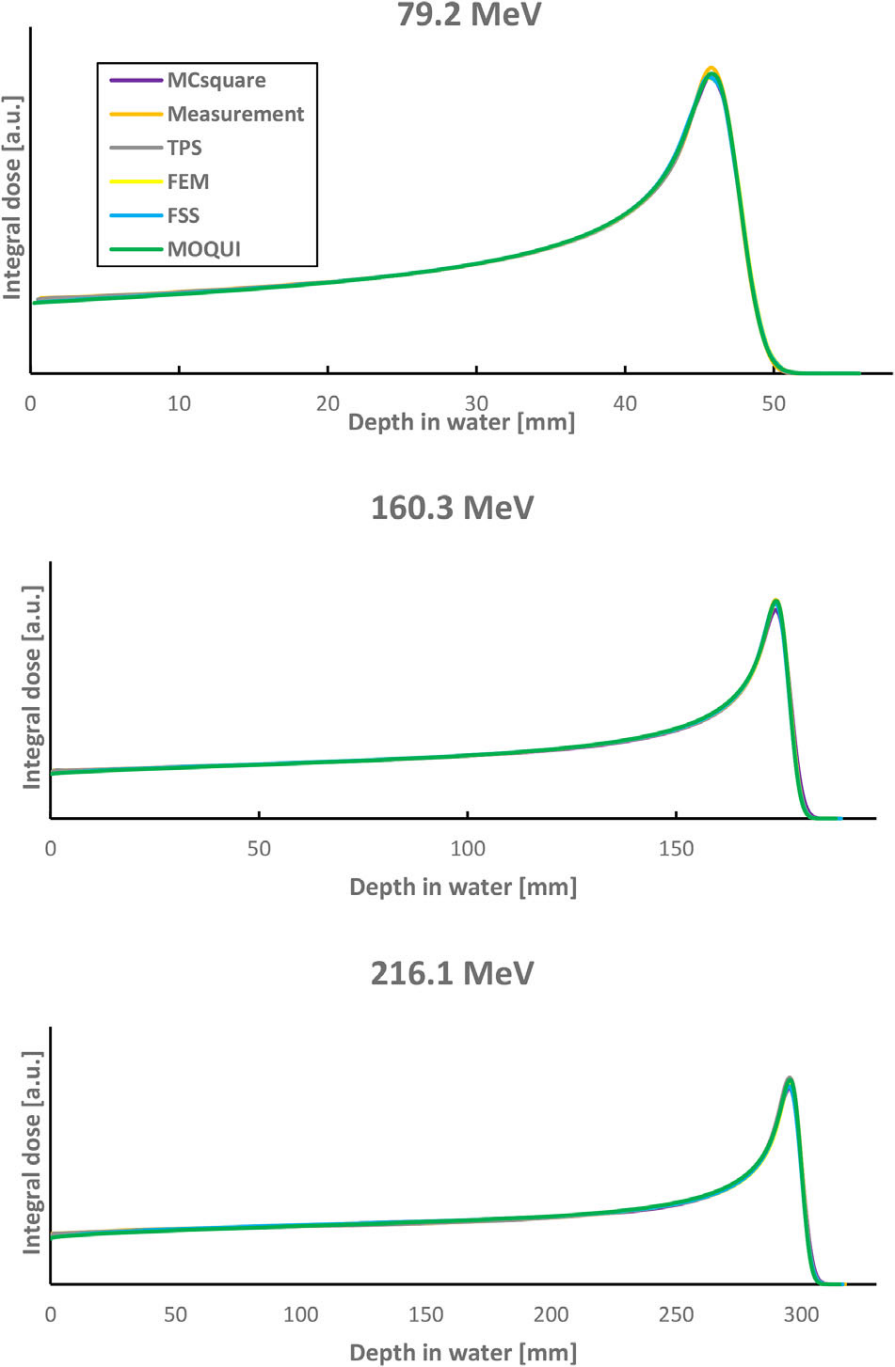
Comparison of TPS, Monte Carlo (MC) codes and measurements of IDDs in a water phantom for protons with different energies.

**FIGURE 2:**
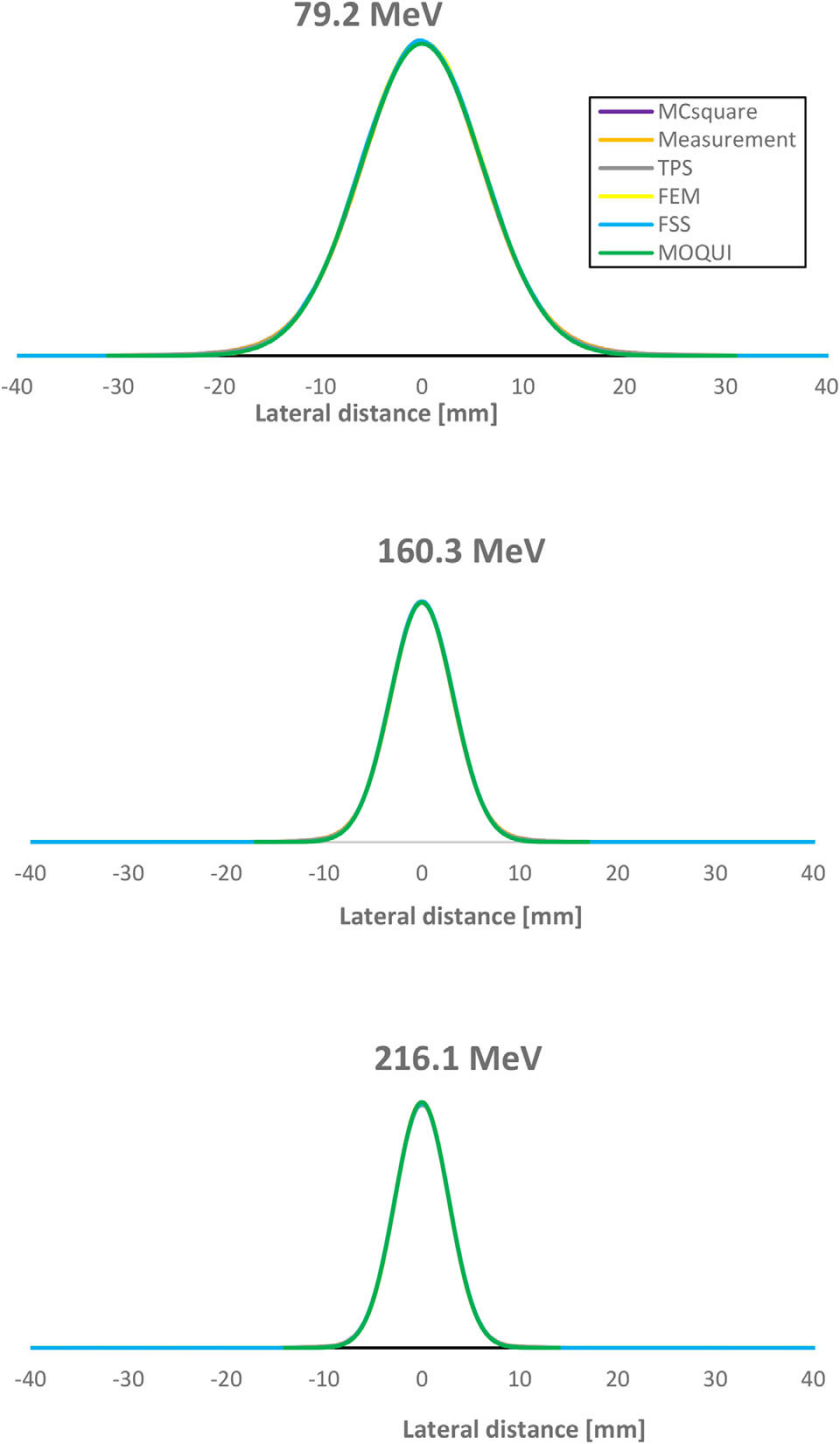
Comparison of TPS, Monte Carlo (MC) codes and measurements of beam profile at isocenter in air for protons with different energies.

Figure 1 presents a comparison of IDDs in water for three monoenergetic proton beams obtained from TPS, MC simulation in all fast MC codes and commissioning measurements. Overall, the IDDs show excellent agreement with minor deviations observed in the Bragg peak region. These differences remain within 1.5% of the peak value for all examined energies. The comparison of measured and simulated lateral propagation of the three proton pencil beams in air at isocenter is shown in Figure 2. The absolute dose verification was performed using the same field size, spot spacing and MUs used in the commissioning procedure with increased simulation histories. All MC codes demonstrated a relative percentage difference of < 0.3% compared with the measurements.

Table 2 summarizes the results of the 3D Gamma Pass Rates (GPRs) of TPS and fast MC codes dose calculation against TOPAS simulations using patient CT for each treatment site. The results indicate consistently high pass rate for the TPS and MCsquare for both 3/3% and 2 mm /2% with the exception of prostate case. While FEM and FSS have very comparable GPRs, the pass rates are lower than TPS and MCsquare. MOQUI GPRs are acceptable for 3 mm/3% criteria, but in the case of 2 mm/2%, they show a relative reduction in pass rate compared with the other MC codes, especially for esophagus, lung, and mediastinum.

**TABLE 2:** 3D Gamma analysis of TPS and fast Monte Carlo (MC) dose calculation versus TOPAS in patient CT.

	3 mm/3%					2 mm/2%				
	TPS	FEM	FSS	MOQUI	MCsquare	TPS	FEM	FSS	MOQUI	MCsquare
Esophagus	99.557	98.244	98.265	97.791	99.648	98.626	97.137	97.196	94.128	97.478
Prostate	99.731	99.234	99.213	99.348	98.789	98.461	98.116	98.162	98.253	94.550
H&N	99.909	98.881	98.866	98.683	99.819	98.547	97.813	97.887	97.469	99.037
Spine	99.857	98.350	98.357	99.055	99.819	98.989	97.912	97.924	98.218	98.987
Brain	99.290	98.631	98.549	98.514	99.371	98.843	98.012	98.032	98.002	98.912
Lung	99.528	98.110	98.010	97.514	99.621	98.968	97.506	97.509	94.161	98.886
Mediastinum	99.231	98.040	98.074	97.359	99.198	98.017	96.644	96.600	94.693	97.559

Table 3 lists the results of the composite fields 2D GPRs of TPS and fast MC codes dose calculation against MatriXX measurements at isocenter across all treatment sites. The observed trends align with those from TOPAS simulations analysis, with TPS and MCsquare having high pass rates, marginally higher than those of FEM and FSS. Notably, MOQUI shows better pass rates for 2 mm/2% criterion.

**TABLE 3:** 2D Gamma analysis of TPS and fast Monte Carlo (MC) dose calculation versus measurements at isocenter.

	3 mm/3%					2 mm/2%				
	TPS	FEM	FSS	MOQUI	MCsquare	TPS	FEM	FSS	MOQUI	MCsquare
Esophagus	98.157	98.597	98.632	97.995	99.080	96.073	96.171	96.219	97.251	97.104
Prostate	98.032	97.101	97.122	98.232	97.567	97.128	97.159	97.198	96.639	97.298
H&N	100.00	99.351	99.392	98.812	99.234	98.910	97.955	98.022	96.401	97.599
Spine	99.120	98.233	98.320	98.703	98.751	96.518	96.082	96.154	95.074	96.325
Brain	98.285	97.224	97.265	98.500	98.151	96.401	95.949	95.982	96.765	96.218
Lung	98.597	98.125	98.132	98.673	98.808	96.753	95.953	96.003	95.697	96.524
Mediastinum	99.081	98.440	98.568	97.132	100.00	98.161	97.861	97.870	97.655	98.405

**FIGURE 3:**
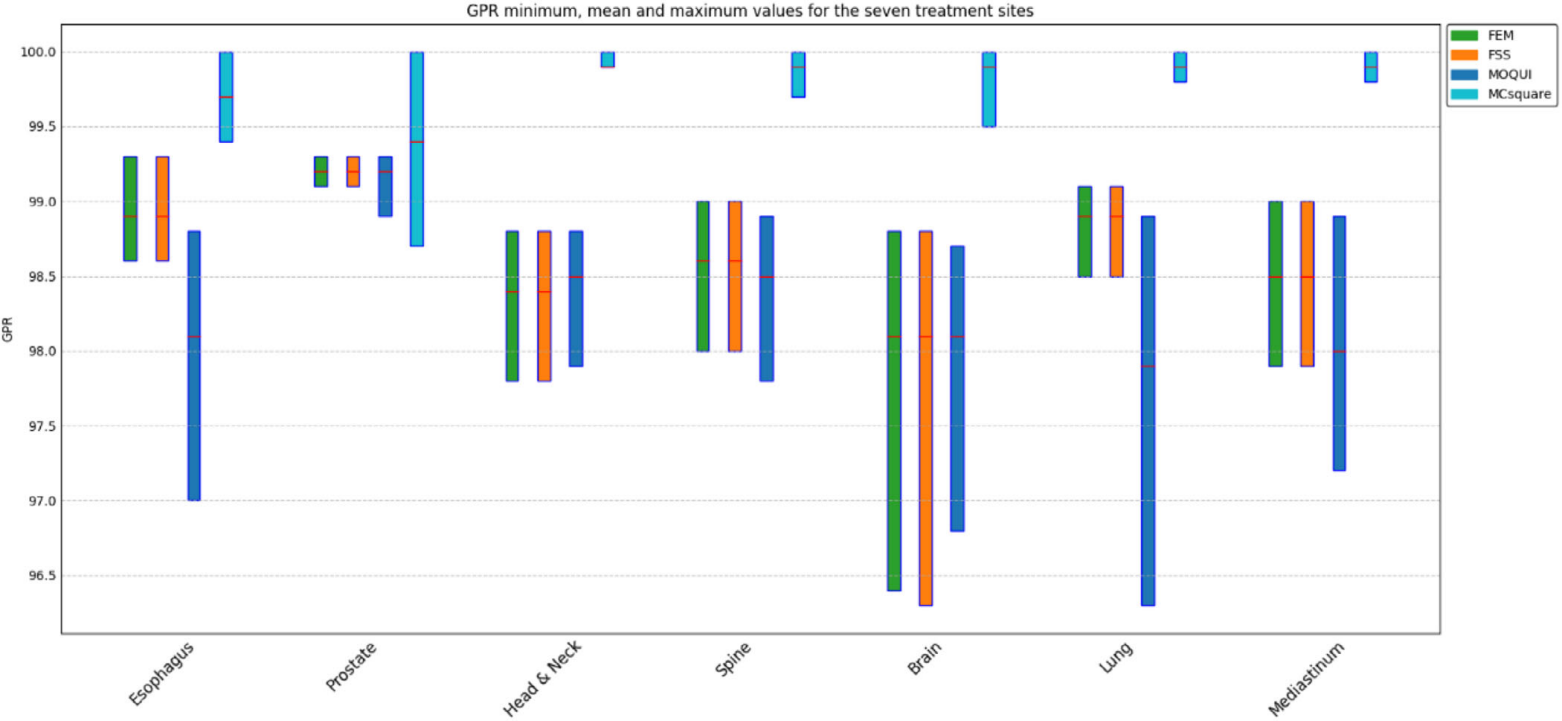
Comparison of the minimum and maximum GPRs for the four MC models. Mean values are indicated in red within each bar.

Figure 3 illustrates the minimum and maximum GPRs for all four MC models compared with the clinical RayStation MC dose engine. For all 70 patients, GPRs were > 96.3%. MCsquare had the highest minimum GPR among the plans for six of the seven disease sites but the lowest minimum GPR among the plans for the prostate. The difference in this lowest GPR from the other three models was about 0.5%. Among the 10 patient plans for the prostate, the overall GPR for MCsquare was higher than that for the other models for seven patients. The minimum GPRs for FEM and FSS were very close. MOQUI had the lowest minimum GPR for plans for four of the seven disease sites. For the maximum GPRs, MCsquare had the highest value (100%) among the plans for all seven disease sites, followed by FEM and FSS, which had almost the same maximum GPRs for all seven sites. MOQUI had the lowest maximum GPR among plans for five of the seven disease sites, and a maximum GPR similar tothose for FEM and FSS for the other two sites. All the MC codes had maximum GPRs > 98.7% for all 70 plans used to compare the models.

**FIGURE 4:**
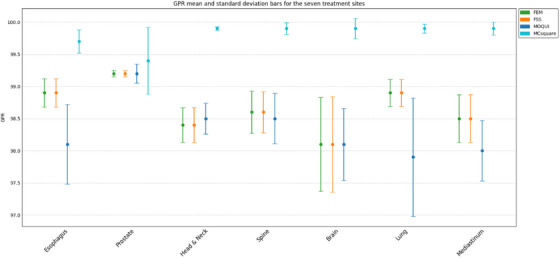
One SD around the mean values of GPR for the four MC models.

MCsquare also had the highest mean GPRs as represented in Figure 4 (range, 99.4%–99.9%) among the plans for all seven disease sites, followed by FEM and FSS, which had almost the same mean GPRs for all seven disease sites. MOQUI had the lowest mean GPRs for plans for four of the seven disease sites, a mean GPR similar to those for FEM and FSS for two sites, and a better mean GPR than that for FEM and FSS for plans for the head and neck. All the models had mean GPRs > 97.9% for all 70 plans used for comparing the models.

It is evident in Figure 4 that MCsquare had the lowest standard deviation (SD) (0.03%–0.18%) among the plans for six of the seven disease sites, followed by FEM and FSS, which had almost the same SD values for all seven disease sites (0.05%–0.74%). MOQUI showed the highest SD for the mean GPR for plans for four of the seven disease sites, a smaller SD than that for FEM and FSS for two sites, and a better SD than that for FEM and FSS for plans for the head and neck.

Table 4 summarizes the mean, standard deviation, sample variance, and 95% confidence interval of GPR for all 70 patients included in this study. Among the MC codes evaluated, MCsquare demonstrated the highest mean GPR, along with the lowest standard deviation and the narrowest confidence interval. Conversely, MOQUI yielded the lowest mean and the highest variability, as reflected by its larger standard deviation and wider confidence interval. FEM and FSS exhibited intermediate performance, with values falling between those of MCsquare and MOQUI.

**TABLE 4:** Mean, SD, sample variance, and confidence level (95%) of the GPR of all 70 patients.

MC code	Mean GPR	SD	Sample variance	Confidence level (95%)
FEM	98.62	0.46	0.21	0.11
FSS	98.62	0.46	0.21	0.11
MOQUI	98.15	0.60	0.36	0.14
MCSquare	99.86	0.15	0.02	0.04

## 4 DISCUSSION

In this study, we commissioned several MC codes to model MD Anderson Cancer Center proton beamline characteristics and compared the resulting models for 70 patients treated in our Proton Therapy Center for seven different sites. The accuracy of dose calculation for all models compared very well with that using RayStation MC dose engine for the different treatment sites plans. The GPRs for the seven treatment sites ranged from 96.3% to 100.0%. Overall, MCsquare, which uses a double Gaussian model, surpassed the other three models in GPR. Nonetheless, it shows some reduction in accuracy, as indicated by its having a lower GPR and higher SD for the mean GPR for the prostate cases than those for the other three models. This may have been caused by the limitation of the model in calculating doses accurately in the presence of high‐energy spots within the plan. FEM and FSS had GPRs ranging from 97.8% to 99.0% for plans for six of the seven disease sites. They had the lowest GPR for brain patients plans. This may have been caused by the limitation of the models in calculating doses accurately in the presence of low‐energy spots in the plan. Also, MOQUI had lower accuracy for the esophagus, lung, and mediastinum cases compared to the other models. This may have been caused by the limitation of the model for medium‐energy spots. The overall high GPR for all four models (> 96%) indicates that the same level of accuracy in dose calculation can be achieved with faster MC codes when compared to more time‐intensive full‐scale MC codes.

In clinical practice, the ability to independently verify treatment plans using efficient and reasonably accurate secondary dose engines is important for ensuring patient safety and maintaining high standards of treatment quality. However, given the absence of widely available commercial secondary dose calculation tools for proton therapy, clinics consider the adoption of reliable and accessible alternatives that can be readily integrated into existing workflows. In this context, comparing the performance of commonly available fast MC dose engines not only offers insight into their relative accuracy and limitations, but also supports their evaluation as potential tools for secondary dose verification in proton therapy.

The results clearly demonstrate the superior performance of MCsquare in reproducing dose distributions closest to those calculated by RayStation, as evidenced by its highest mean GPR and lowest standard deviation. However, it is important to acknowledge that MCsquare employs a double 2D Gaussian beam model, whereas the other codes utilize a single 2D Gaussian model. This difference in beam modeling is likely a significant contributing factor to the observed higher accuracy of MCsquare. To enable a more equitable and comprehensive comparison across all codes, it would be beneficial if future versions of FRED and MOQUI incorporate support for double Gaussian beam modeling.

Another important aspect of this work is the difference in computational time for the MC models. Even though every MC code runs on different platforms that are hard to compare, especially when comparing CPU and GPU architectures, highlighting the average simulation time for each code is crucial. Using MD Anderson High Performance Computing for Research resource, we ran MCsquare on 80 CPUs, with an average computational time of 5–8 min per patient. On the other hand, for MOQUI, the average time was 3–5 min per patient using a single GPU device. The average execution time for FEM and FSS was 1–2 min, as they can run on four GPU devices and exhibits near‐linear performance scaling.

It is important to emphasize that the primary objective of the comparison, presented in Table 2—between TPS and fast MC codes versus TOPAS is to serve as a benchmarking and validation for the fast MC codes. The fact that GPR of one code is comparable to that of another code does not imply that an intercomparison between the two codes will yield similar results. The results of such comparisons are neither automatically nor strictly transitive, owing to the inherent complexity and spatial sensitivity involved in 3D gamma analysis. Therefore, comparing each fast MC code to a full‐scale MC simulation provides an essential and direct benchmarking reference to ensure acceptable performance and reliability in clinical applications.

The findings of this work must be seen in light of some limitations. An important limitation of our study is related to the physics models implemented in the MC codes that we used,^15^, ^19^, ^22^ especially for secondary particles, as prompt gammas, electrons, and neutrons are not transported, which affects the dose calculation accuracy to some extent.

## 5 CONCLUSION

All commissioned fast MC codes examined in this study demonstrated acceptable 3D GPRs for all tested patients and treatment sites. Even though MCsquare was commissioned using a double 2D Gaussian model, the single 2D Gaussian model used in the two GPU codes proved to be adequate and resulted in GPRs at the high end of the pass rate. In fact, especially when using multiple GPU devices, the nonnegligible reduction in GPU MC running time for FEM and FSS justifies the relative decrease in GPR as compared to MCsquare.

## AUTHOR CONTRIBUTIONS

All authors contributed to the conception of the work. Sherif M. Gadoue ran the simulations, generated data, and wrote parts of the manuscript. Narayan Sahoo evaluated the results, performed data analysis, and wrote parts of the manuscript. All authors approved the final version of the manuscript.

## CONFLICT OF INTEREST STATEMENT

The authors declare no conflicts of interest.
